# Silicon via fertigation with and without potassium application, improve physiological aspects of common beans cultivated under three water regimes in field

**DOI:** 10.1038/s41598-024-52503-8

**Published:** 2024-01-24

**Authors:** Carlos Vital Gonzalez-Porras, Gelza Carliane Marques Teixeira, Renato de Mello Prado, Patrícia Messias Ferreira, Luiz Fabiano Palaretti, Kamilla Silva Oliveira

**Affiliations:** 1 Department of Agricultural Sciences, São Paulo State University (UNESP), Jaboticabal, São Paulo, Brazil; 2 Department of Engineering and Exact Sciences, São Paulo State University (UNESP), Jaboticabal, São Paulo, Brazil

**Keywords:** Plant physiology, Plant stress responses

## Abstract

Frequent droughts have led to an expansion of irrigated common bean (*Phaseolus vulgaris* L.) cultivation areas. An effective strategy to enhance water use efficiency and optimize crop growth is the application of silicon (Si) and potassium (K). However, the interaction between Si dosage, water regimes, and plant potassium status, as well as the underlying physiological mechanisms, remains unknown. This study aimed to assess the effects of Si doses applied via fertigation under various water regimes, in the presence and absence of potassium fertilization, on gas exchange, water use efficiency, and growth of Common beans in field conditions. Two experiments were conducted, one with and one without K supply, considering that the potassium content in the soil was 6.4 mmol_c_ dm^-3^ in both experiments and a replacement dose of 50 kg ha was applied in the with K treatment, with the same treatments evaluated in both potassium conditions. The treatments comprised a 3 × 4 factorial design, encompassing three water regimes: 80% (no deficit), 60% (moderate water deficit), and 40% (severe water deficit) of soil water retention capacity, and four doses of Si supplied via fertigation: 0, 4, 8, and 12 kg ha^−1^. Where it was evaluated, content of photosynthetic pigments, fluorescence of photosynthesis, relative water content, leaf water potential and electrolyte extravasation, dry mass of leaves, stems and total. The optimal doses of Si for fertigation application, leading to increased Si absorption in plants, varied with decreasing soil water content. The respective values were 6.6, 7.0, and 7.1 kg ha^−1^ for the water regimes without deficit, with moderate water deficit, and with severe water deficit. Fertigation application of Si improved plant performance, particularly under severe water deficit, regardless of potassium status. This improvement was evident in relative water content, leaf water potential, and membrane resistance, directly impacting pigment content and gas exchange rates. The physiological effects resulted in enhanced photosynthesis in water-deficient plants, mitigating dry mass production losses. This research demonstrates, for the first time in common bean, the potential of Si to enhance irrigation efficiency in areas limited by low precipitation and water scarcity.

## Introduction

The continuous growth of the global population and increased land use for agriculture have intensified the demand for water resources^1^. Currently, approximately 75% of the world's water consumption is attributed to agricultural production^2^. However, around 35% of water is lost due to inefficiencies in irrigation system infrastructure or distribution^3–5^.

The availability of water in agricultural systems is increasingly affected by climate change, characterized by rising temperatures, reduced rainfall, and prolonged droughts^6,7^. It is projected that by 2050, the demand for irrigation water will increase by 11%^8,9^, Consequently, future scenarios indicate potential conflicts arising from increased competition for water for irrigation especially in areas with decreased water resources^10^.

Therefore, implementing strategies to enhance water use efficiency and encourage responsible utilization of this vital resource is crucial. Balanced nutrition represents a promising approach for sustainable irrigated agriculture, utilizing elements and nutrients with the potential to increase water use efficiency, such as potassium (K) and silicon (Si)^11^. K is an essential nutrient that contributes to osmotic adjustment, stomatal regulation, and transpiration rate in plants, thereby enhancing water use efficiency^12^. Si, on the other hand, is a beneficial element known for its ability to mitigate damage caused by water deficit^13–16^.

The beneficial effects of Si in alleviating damage caused by water deficit have been demonstrated in several species, particularly in the Poaceae group, including maize^15,17^, pre-sprouted sugarcane seedlings^18^, sugar cane stumps^16^, and forage plants^19^. However, the effectiveness of Si is influenced by a plant's ability to absorb and accumulate it^20^. As a result, most Si studies have focused on plants that naturally accumulate this element, such as *Poaceae* species, which possess active absorption mechanisms through transporters^21^. Scientific evidence on plants that passively absorb Si, primarily driven by the transpiration gradient, such as common beans, is still limited^22^. Specific studies on common beans cultivated in hydroponic systems have shown that Si improves potassium absorption, resulting in increased photosynthetic rates, higher levels of photosynthetic pigments, reduced oxidative stress, and enhanced water use efficiency^23^. However, in the fava bean, which belongs to the (Fabaceae) family, Si alleviates and improvement the damage caused by water deficit in gas exchange, fluorescence and the water content in tissues^24^.

The increased application of Si in field conditions and the limitations posed by the low potential Si uptake in non-accumulating plants can be addressed by improving application techniques. Fertigation of Si has the potential to enhance Si absorption by utilizing high-quality solutions with low Si polymerization rates. Polymerization is minimized when Si concentrations in the solution are kept below 3.0 mmol L^−1^, ensuring the presence of monomeric forms of Si (H_4_SiO_4_) that can be absorbed by plants^25^. The use of a soluble Si source adequately stabilized with sorbitol further helps maintain the stability of monomeric Si forms^26^. These factors, combined with frequent application, facilitate consistent Si absorption and accumulation in plants. However, this approach needs to be studied in non-accumulating plants, such as common beans, under field conditions.

Considering the potential enhancement of irrigated common bean cultivation, supplying Si via fertigation represents a viable option for promoting sustainable plant growth. However, there is a dearth of research on this particular species aiming to understand the physiological mechanisms induced by Si and whether the optimal Si dosage varies under different water regimes or with varying K status in plants. Therefore, our hypothesis are: (i) Si's beneficial effects on common beans in field studies may enhance gas exchange by increasing photosynthetic pigments, thus improving water use efficiency and dry mass conversion; (ii) these benefits of Si may manifest in both water-deficient and water-sufficient regimes; (iii) a higher Si dosage may be required in water-deficient regimes due to reduced Si absorption compared to the water-sufficient regime, regardless of K status.

If these hypotheses hold true, it will provide valuable insights into the physiological mechanisms underlying the growth of common bean crops induced by Si. This knowledge can then be utilized to implement precise technologies based on optimal Si dosages in regions with abundant water resources and those facing water scarcity, regardless of the plant's K status. This study will demonstrate, for the first time, that Si's benefits extend beyond stress-induced conditions, as often emphasized in the literature for other crops^27^. The advantages of Si in stress-free field production systems contribute to enhancing water use efficiency and the sustainability of irrigated common bean cultivation systems, which is of global significance given the widespread impact of climate change and water restrictions on areas cultivating this particular species.

This research aims to evaluate the effects of Si doses applied via fertigation under different water regimes, including moderate and severe water deficits, in the presence and absence of potassium fertilization, on gas exchange, water use efficiency, and the growth of common bean crops under field conditions.

## Material and methods

### Growing conditions and plant material

**Figure 1:**
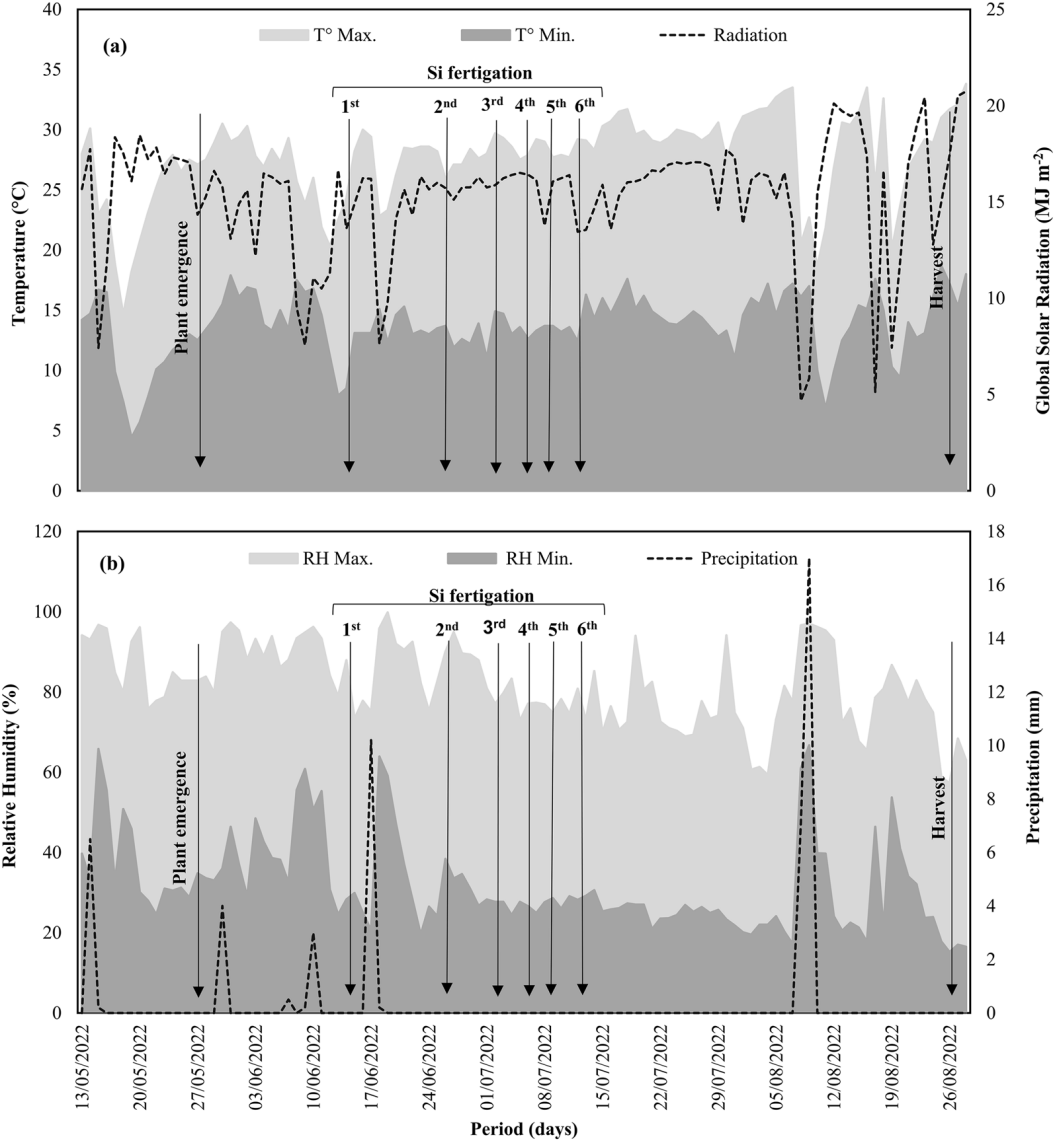
Experimental conditions in the area where the field experiment was conducted. Maximum temperature (T° Max), minimum temperature (T° Min), global radiation (**a**), maximum relative humidity (RH Max), minimum relative humidity (RH Min), and rainfall (P) (**b**).

This research was conducted on common bean crops under field conditions at the Teaching Farm, Research and Extension site of São Paulo State University, located in Jaboticabal, São Paulo, Brazil (latitude: − 21º14′51.57"; longitude: − 48º17′02.07"; altitude: 546 m). The experiment was conducted from May to August 2022, and meteorological data including air temperature, relative humidity, global radiation and rainfall in the experimental area were recorded (Fig. 1), which were important to determine the irrigation sheets. The meteorological elements used in this work were extracted from a set of data belonging to the collection of the agrometeorological area of ​​the department of exact sciences. The observations made at the agroclimatic station on the Jaboticabal campus are collected, typed in a standardized format, consistency and quality control are carried out. The monthly and annual daily averages are then obtained and passed on to users.

The soil in the experimental area was classified as Eutroferric Red Latosol^28^ corresponding to Oxisol in the American classification system (Soil Taxonomy). Before the experiment, soil samples were collected from the 0–20 cm and 20–40 cm depths for chemical analysis (Table 1), to determine soil fertility purposes^29–31^. Available Si content was 3.0 mg dm^−3^ and it was considered as low in the soil^32^. Particle size analysis of the soil was performed^33^. The results of the soil chemical analysis at the 0–20 cm and 20–40 cm depths were as follows:

**Table 1:** Chemical attributes of only beds of 0–20 and 20–40 cm depth.

Prof	pH	OM	P	S	Ca	Mg	K	Al	H + Al	SB^a^	T^b^	V^c^	m^d^	B	Cu	Fe	Mn	Zn
cm	CaCl_2_	g dm^−3^	mg dm^−3^		mmol_c_ dm^−3^							%		mg dm^3^				
0–20	6.2	20.5	59	11	37	17	6.2	1	22.5	60.6	82.9	73	1	0.41	6.4	11	23.6	3.9
20–40	5.6	19	36	13	40	18	6.4	0	23	64	87.2	73	0	–	–	–	–	–

^a^Sum of bases (SB = Ca^2+^  + Mg^2+^  + K^+^).

^b^Cation exchange capacity (T = SB + H + Al).

^c^Basis saturation (V = SBx100/T).

^d^Aluminum saturation.

Soil preparation in the experimental area followed a conventional planting system, involving subsoiling and harrowing with heavy and light harrows.

The common bean cultivar used in this research was Carioca BSR FC 402. Seeds were collected from public lands, being granted by the Brazilian Agricultural Research Corporation of the Ministry of Agricultural, Livestock and Food Supply, Brazil. All plant studies were carried out following relevant institutional, national, or international guidelines and regulations. Our research was not conducted with endangered species and was conducted following the Declaration of IUCN Policy on Research Involving Endangered Species.

The plants had a normal cycle of 85 to 94 days (from emergence to maturation), known for its high productivity, nutritional value, and resistance to pests and diseases^34^. The planting density was set at 333,000 plants per hectare.

### Treatments and experimental design

Two experiments were conducted in the same common bean cultivation area, analyzing two different potassium (K) status. Each experiment followed a 3 × 4 factorial design, with three water regimes: 80% of soil water retention capacity (WRC) (without water deficit) (WWD), 60% WRC (moderate water deficit) (MWD), and 40% WRC (severe water deficit) (SWD), and four doses of Si supplied via fertigation: 0, 4, 8, and 12 kg ha^−1^. The treatments were evaluated under two potassium conditions: without fertilization (− K) in Experiment 1 and with fertilization (+ K) in Experiment 2; with a potassium content in the soil 6.4 mmol_c_ dm^-3^ in both experiments. The experiments were set up using a sub-divided plot design within a randomized block design, with four replications. The experimental plots were 2.25 m wide, with row spacing of 0.45 m and a length of 6 m. The total area was 13.5 m^2^, with an evaluation area of 5.4 m^2^ (Supplementary Fig. 1).

The Si doses were determined based on the recommendations of the International Rice Research Institute (IRRI), which suggest using doses of 40 to 60 kg ha^-1^ of potassium silicate (15% Si) (equivalent to 6 to 9 kg ha^−1^ of Si)^35^. The doses were applied in six applications at 22, 32, 38, 41, 44, and 47 days after full emergence (DAE). The applied doses were divided according to the number of applications, resulting in concentrations of 0, 0.67, 1.33, and 2.00 kg ha^−1^ of Si. These concentrations corresponded to 0, 0.96, 1.90, and 2.85 mmol L^−1^ of Si in the solution for each application. The splitting of doses was performed to promote increased Si absorption, ensuring that the concentrations in each application remained below 3.5 mmol L^−1^ of Si, thus minimizing the risk of Si polymerization^25^.

Sodium silicate stabilized with sorbitol (Si = 115.2 g L^−1^, Na_2_O = 60.5 g L^−1^) was used as the Si source in both experiments. The sorbitol in this source has stabilizing properties that help maintain higher concentrations of monomeric Si forms^26^, reducing the risk of Si polymerization in the solution.

Fertilization was carried out based on soil nutrient content and the recommendations for common bean crops^36^. Phosphate fertilization involved applying a dose of 40 kg ha^−1^ of P2O5 in the form of simple superphosphate in the sowing furrows. Nitrogen fertilization was applied via fertigation, with urea split into four applications of 20, 30, 45, and 45 kg ha^−1^ of N at 7, 20, 31, and 43 DAE, respectively. In plots with K fertilization, a dose of 50 kg ha^−1^ of K_2_O in the form of KCl was applied manually at 14 DAE. In plots without potassium fertilization, no K was applied, but other nutrients were supplied as recommended.

Phytosanitary management included control of the screwworm (*Agrotis ipsilon*) at 6 DAE using Chlorantraniliprole and Lambda-cyhalothrin (250 mL) + Mineral oil (150 mL adjuvant) in 150 L of syrup, applied at a rate of 335 L ha^−1^. A preventive application to control whitefly (*Bemisia tabaci*) and fungi was performed at 14 DAE using Thiamethoxam (100 g L^−1^) + Fluxapyroxad and Pyraclostrobin (150 mL) + Mineral oil (150 mL) in 120 L of syrup, applied at a rate of 250 L ha^−1^. Applications to control whitefly were also carried out at 30 and 42 DAE using Thiamethoxam (100 g L^−1^) + Mineral oil (150 mL) in 120 L of syrup, applied at a rate of 250 L ha^−1^. Weed control was performed manually until the rows were closed by the plants.

A self-compensating drip irrigation system was installed in the experimental area, with emitters spaced at 0.5 m intervals and a flow rate of 1.6 L h^−1^ (Dripnet PC 16,250). Each crop line was equipped with a drip line. Water holding capacity levels in the soil (WRC) were determined by collecting undisturbed soil samples. The water retention curve was constructed through tests on a tension table and Richard’s pressure chamber^37^. From the water retention curve, the field capacity (FC) was determined to be 0.41 cm^3^ cm^−3^, and the permanent wilting point (PWP) was determined to be 0.13 cm^3^ cm^−3^. Using these values, the available water (AW) was calculated as 0.28 cm^3^ cm^−3^, and consequently, the total available water capacity (TAW) was determined using AW and the effective root system of the common bean plants, which was 0.4 m. TAW was calculated to be 112 mm. Based on the TAW value, the soil water retention capacity levels (WRC) were adjusted to 89.6 mm, 67.2 mm, and 44.8 mm for the water regimes of 80%, 60%, and 40% of WRC, respectively.

Initially, the plants were maintained under adequate water conditions (80% of WRC), while moderate and severe water deficit levels were imposed during the pre-flowering period (R5). The water volume applied to maintain the SWC levels was determined through daily water balance calculations, taking into account the excess or deficit of water in the soil concerning the WRC of each water regime. The inputs considered in this system included irrigation water and rainfall, while the only water output considered was crop evapotranspiration (ETc), assuming that deep percolation and runoff were negligible^38^.

During the experiment, the volumetric soil moisture (θ) was monitored daily using the Time Domain Reflectometer (TDR) method (HydroSenceII), which is an indirect method. For TDR use, soil samples were initially collected at the same points to determine the soil moisture directly based on mass. The TDR's precision with soil moisture was considered sufficient with an R^3^ = 0.84 (y = 1.0294x—3.2562), where the TDR readings underestimated the soil moisture (θ) by 2.2% with a standard deviation of 1%. The established soil moisture levels for each water regime were 0.354, 0.283, and 0.212 cm^3^ cm^−3^ for the water regimes of 80%, 60%, and 40% of WRC, respectively.

The irrigations were applied when the water deficit reached the maximum allowable depletion of available water in the soil. For the irrigation scheme recommended by FAO, i.e., under the condition without water deficit, the experimental plots were irrigated when 45% of the available water in the soil was depleted, resulting in a soil moisture (θ) of 0.2864 cm^3^ cm^−3^ and water storage of 49.58 mm. The depletion factors for the moderate and severe deficit treatments were 60% and 80%, with soil moisture (θ) at 0.242 and 0.184 cm^3^ cm^−3^ and water storage of 26.88 and 13.44 mm, respectively^38^.

The plants were cultivated throughout the entire phenological cycle, and morphological. Physiological, and nutritional analyses were carried out during the pod formation stage (R7). The analyses were determined by collecting samples from 10 plants per plot, collected within the useful area.

### Analyses

#### Fluorescence and quantum efficiency of the photosystem II (Fv/Fm)

Initial (Fo) and maximum (Fm) fluorescence, as well as photosystem II quantum efficiency (Fv/Fm), were determined on the central leaflet of the fifth fully developed leaf. Readings were taken between 7:00 am and 9:30 am using a portable fluorometer (Os30P +, Opti-Sciences Inc., USA)^39^. Prior to the readings, the leaves were kept in the dark for 30 min. This variable was measured 64 DAE.

#### Chlorophyll and carotenoid content

Five leaf discs measuring 26.4 mm^2^ each were collected from the middle third of the leaf blade of the fourth fully developed leaf. The fresh mass of each disc was immediately determined by weighing. The samples were then depigmented in 80% acetone, and readings were taken using a spectrophotometer (DU640, Beckman, USA) at wavelengths of 663 nm for chlorophyll a (Chl*a*), 647 nm for chlorophyll b (Chl*b*), and 470 nm for carotenoids^40^. The results are expressed in mg g^-1^ of fresh plant material. This variable was measured 65 DAE.

#### Leaf gas exchange

Net photosynthesis (A), transpiration rate (E), stomatal conductance (Gs), and internal carbon concentration (Ci) were measured using an open infrared gas analyzer (IRGA) (LcPro-SD, ADC BioScientific Ltd., RB). The IRGA chamber was exposed to a photosynthetic photon flux density of 1,200 μmol m^−2^ s^−1^ on the central leaflet of the fifth fully developed leaf^23,41^. Readings were taken between 9:00 am and 11:00 am. Intrinsic water use efficiency (WUE = A/E) was calculated based on the relationship between A and E. The instantaneous efficiency of carboxylation (EIC = A/Ci) was determined by the ratio of A to Ci. These measurements were only taken in plants that did not receive potassium fertilization. This variable was measured 80 DAE.

#### Relative water content

Ten leaf discs measuring 26.4 mm^2^ each were collected from the central leaf of fifth trifoliate fully developed. The discs were immediately weighed to obtain the fresh mass (Mf). Subsequently, the samples were rehydrated in deionized water for six hours to obtain the turgid mass (Mt). The samples were then dried in an oven with forced air circulation at 80 °C for 24 h to obtain the dry mass (Md). The relative water content was calculated using the formula [(Mf − Md)/(Mt − Md)] × 100^42^. This variable was measured 65 DAE.

#### Leaf water potential (Ψw)

Leaf water potential was determined by evaluating the fully developed fifth leaf using a Scholander pressure chamber (3000F01, Soil Moisture Equipment, USA). Pressure was applied until exudation occurred at the cut area of the leaf. Measurements were taken between 5:00 am and 7:00 am^43^. This variable was measured 70 DAE.

#### Electrolyte leakage index

Ten leaf discs measuring 26.4 mm^2^ each were collected from the fourth fully developed leaf and immersed in deionized water for two hours. The electrical conductivity (EC1) of the solution was then measured using a conductivity meter (AK51, Akso, BR). The samples were subsequently autoclaved at 121 °C for 20 min, and after cooling, a new electrical conductivity reading (EC2) was taken. The electrolyte leakage index was determined using the formula: EC1/EC2 × 100^44^. This variable was measured 65 DAE.

#### Dry mass production

Plant shoots were collected, separated into leaves and stems, and then washed with running water, followed by a detergent solution (0.1% v/v), HCl solution (0.3% v/v), and finally deionized water. The plant material was then dried in a forced air circulation oven (TE-394/3-MP, Tecnal, BR) at 65 ± 5 °C until a constant mass was achieved from where they were weighed on a precision analytical balance. This variable was measured 71 DAE.

#### Leaf analysis of Si and K

The samples collected to determine the dry mass production were first ground using a Willey-type mill, which combined both leaves and stems. The samples were dried and ground in a Willey-type mill. The Si content was extracted using hydrogen peroxide and sodium hydroxide solution^45^. The samples were then measured using a spectrophotometer (B442, Micronal, BR) at a wavelength of 410 nm to determine Si content^32^.

Leaf K content was determined through perchloric and nitric acid digestion. The reading was obtained using atomic absorption spectrophotometry with an air-acetylene flame^46^. Based on the Si and K contents in the plant's dry mass, the accumulation of Si and K was calculated using the Eq. (1).

1 $${\varvec{Accumulation\,of\,Si\,or\,K}}\left({\varvec{mg\,per\,plant}}\right)=shoot\, dry\, mass\, (g\, per\, plant)\times Si\, or\, K \,content \left(mg\, {kg}^{-1}\right)$$

### Statistical analysis

The data were subjected to analysis of variance using the F-test (p ≤ 0.05) after verifying normality and homoscedasticity of variances (Shapiro–Wilk W test and Bartlett test). Quantitative data, corresponding to Si doses, were analyzed using polynomial regression models. Qualitative data, corresponding to water regimes, were analyzed using the Tukey mean comparison test (*p* ≤ 0.05).

The difference between treatments was also analyzed using a hierarchical cluster analysis, where the Euclidean distance was used as the coefficient of similarity. Additionally, a correlation network was used to graphically express the functional relationship between the estimates of Pearson correlation coefficients among the variables, where the proximity between nodes (traits) was proportional to the absolute value of the correlation between those nodes. Positive correlations were highlighted in green, while negative correlations were represented in red. The statistical analyses were conducted using the R programming language (version 4.3.1, R Core Team).

### Statement of handling of plants

Seeds were collected from public lands, being granted by the Brazilian Agricultural Research Corporation of the Ministry of Agricultural, Livestock and Food Supply, Brazil. All plant studies were carried out following relevant institutional, national, or international guidelines and regulations. This research was not conducted with endangered species and was conducted in accordance with the Declaration of IUCN Policy on Research Involving Endangered Species.

## Results

### Silicon and potassium accumulation

**Figure 2:**
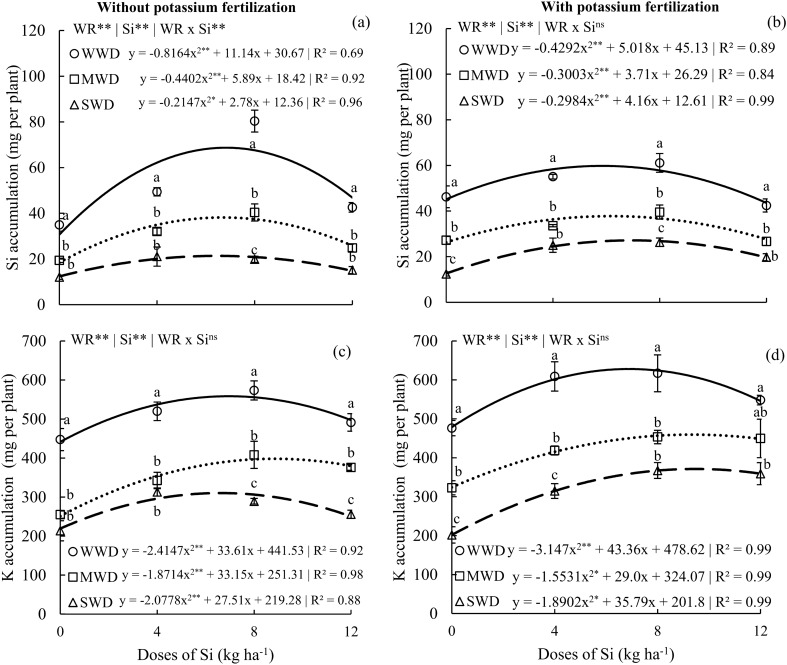
Accumulation of silicon (Si) (**a**,**b**) and potassium (K) (**c**,**d**) in the shoots of common bean plants cultivated without water deficit (WWD, 80% of the water retention capacity—WRC), with moderate water deficit (MWD, 60% of WRC), and with severe water deficit (SWD, 40% of WRC), the plants were subjected to four doses of Si (0, 4, 8, and 12 kg ha^−1^) via fertigation, with and without potassium fertilization. Letters indicate significant differences among water regimes (WR) at each Si dose (p < 0.05, Tukey’s test). * and **Indicate significance at the 1% and 5% probability levels, respectively, while ns indicates no significance based on the F test.

The accumulation of silicon (Si) in plants without potassium (K) fertilization showed interactive effects between water regimes and Si doses (p < 0.01) (Fig. 2a). In plants with potassium fertilization, there was only an isolated effect of the factors water regime and Si doses (p < 0.01) (Fig. 2b). Si doses resulted in Si accumulation following a quadratic polynomial adjustment in the three water regimes and in both K conditions. In plants without potassium fertilization, the maximum Si accumulations were 68.6, 38.1, and 21.3 mg per plant of Si, obtained with doses of 6.8, 6.7, and 6.5 kg Si ha^-1^, respectively, for WWD, MWD, and SWD (Fig. 2a). Plants with potassium fertilization showed a maximum accumulation of Si of 59.7, 37.7, and 21.3 mg per plant, obtained at doses of 5.8, 6.1, and 6.9 kg Si ha^-1^, respectively, for WWD, MWD, and SWD (Fig. 2b). The lowest Si accumulation was observed in the MWD and SWD regimes at all Si doses evaluated in relation to the WWD condition, regardless of the K condition.

The factors water regime and Si doses affected the accumulation of potassium (K) in plants in both conditions of potassium fertilization in an isolated manner (p < 0.01). Si doses resulted in a quadratic polynomial fit for all water regimes (Fig. 2c,d). Plants without K fertilization showed maximum K accumulations of 558.4, 398.1, and 310.3 mg per plant at Si doses of 6.9, 8.8, and 6.6 kg ha^−1^, respectively. The maximum accumulations of K in plants that received potassium fertilization were 627.9, 459.4, and 371.2 mg per plant at doses of 6.8, 9.3, and 9.4 kg Si ha^−1^, respectively, for the WWD, MWD, and SWD regimes. No Si supply (0 kg ha^−1^) in plants under the MWD and SWD water regimes showed the lowest K accumulations without potassium fertilization and with potassium fertilization in relation to the WWD condition. However, without K fertilization, the doses of 8 and 12 kg Si ha^−1^ showed a distinction between the regimes regarding the accumulation of K (Fig. 2c), while with potassium fertilization, this occurred at the doses of 0 and 4 kg Si ha^−1^ (Fig. 2d).

### Silicon effects in plants hydric status

**Figure 3:**
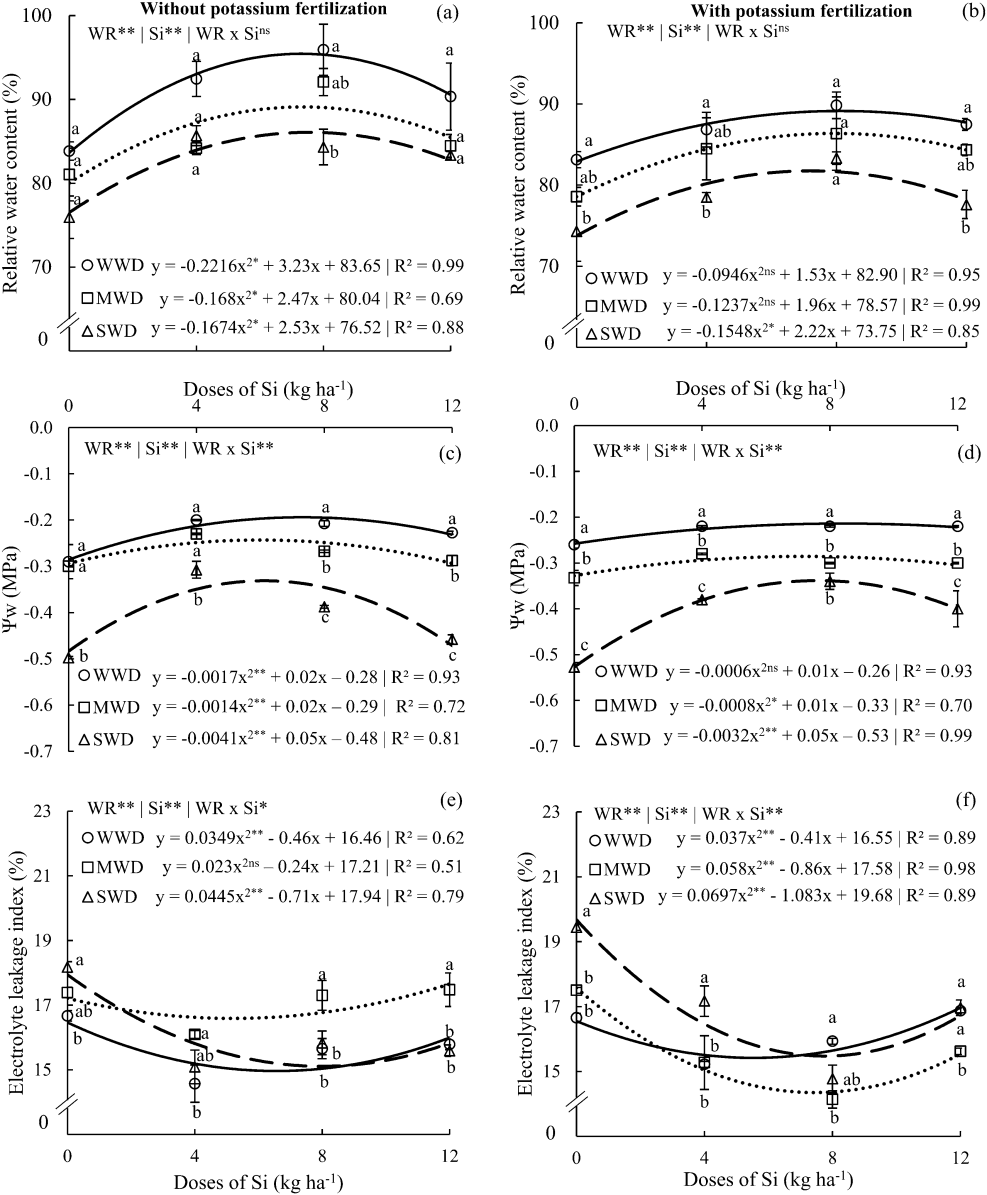
Relative water content (**a**,**b**), water potential (Ψw) (**c**,**d**), and electrolyte leakage index (**e**,**f**) in common bean plants cultivated without water deficit (WWD, 80% of the water retention capacity—WRC), with moderate water deficit (MWD, 60% of WRC), and with severe water deficit (SWD, 40% of WRC), the plants were subjected to four doses of Si (0, 4, 8, and 12 kg ha^−1^) via fertigation, with and without potassium fertilization. Letters indicate significant differences among water regimes (WR) at each Si dose (p < 0.05, Tukey’s test). * and **Indicate significance at the 1% and 5% probability levels, respectively, while ns indicates no significance based on the F test.

The relative water content and water potential in plants without potassium fertilization and with potassium fertilization showed effects only for the isolated factors (water regime and Si dose) (all at p < 0.01), with a quadratic polynomial fit for all water regimes evaluated (Fig. 3a–d). The maximum water contents (95.4, 89.1, and 86%) were observed in plants without potassium fertilization at Si doses of 7.3, 7.4, and 7.6 kg ha^−1^ for WWD, MWD, and SWD, respectively (Fig. 3a). For plants with potassium fertilization, doses of Si at 8.0, 7.9, and 7.1 kg ha^−1^ provided the maximum water content (89.0, 86.3, and 81.7%) (Fig. 3b). Without potassium fertilization, plants under WWD showed higher water contents only at the dose of 8 kg Si ha^−1^ in relation to plants under SWD. However, with potassium fertilization, this was observed at the doses of 0, 4, and 12 kg Si ha^−1^.

The water potential showed interaction effects (p < 0.01) between water regimes and Si doses without potassium fertilization and with potassium fertilization. The maximum leaf water potentials in plants without K fertilization were − 0.22, − 0.21, and − 0.32 MPa at Si doses of 5.8, 7.1, and 6.0 for the WWD, MWD, and SWD water regimes, respectively (Fig. 3c). However, in plants with potassium fertilization, Si doses of 8.3, 6.2, and 7.8 kg ha^−1^ provided the maximum leaf water potential (− 0.21, − 0.29, and − 0.33 MPa, respectively) (Fig. 3d). In both K conditions, plants under SWD showed lower water potentials at all Si doses. However, plants without potassium fertilization in the MWD water regime that received a dose of 4 kg Si ha^−1^ showed a water potential equal to that of plants under WWD.

The extravasation of electrolytes in plants without potassium fertilization and with potassium fertilization showed interaction effects (WR x Si) (p < 0.05 and p < 0.01, respectively), with a decreasing quadratic polynomial fit (Fig. 3e,f). The minimum extravasation of electrolytes without potassium fertilization occurred at Si doses of 6.6, 5.2, and 8.0 kg ha^−1^, with values of 14.9, 16.5, and 15.1% for the WWD, MWD, and SWD water regimes, respectively (Fig. 3e). Plants with potassium fertilization showed the least leakage (15.4, 14.3, and 15.4%) at doses of 5.5, 7.4, and 7.7 kg Si ha^−1^ (Fig. 3f). The SWD in both K conditions showed greater leakage at the dose of 0 kg Si ha^−1^. However, at a dose of 8 kg Si ha^−1^ without potassium fertilization, plants under SWD and WWD showed less electrolyte leakage in relation to MWD; in plants with potassium fertilization, this occurred in relation to SWD.

### Silicon effects in photosynthesis parameters

**Figure 4:**
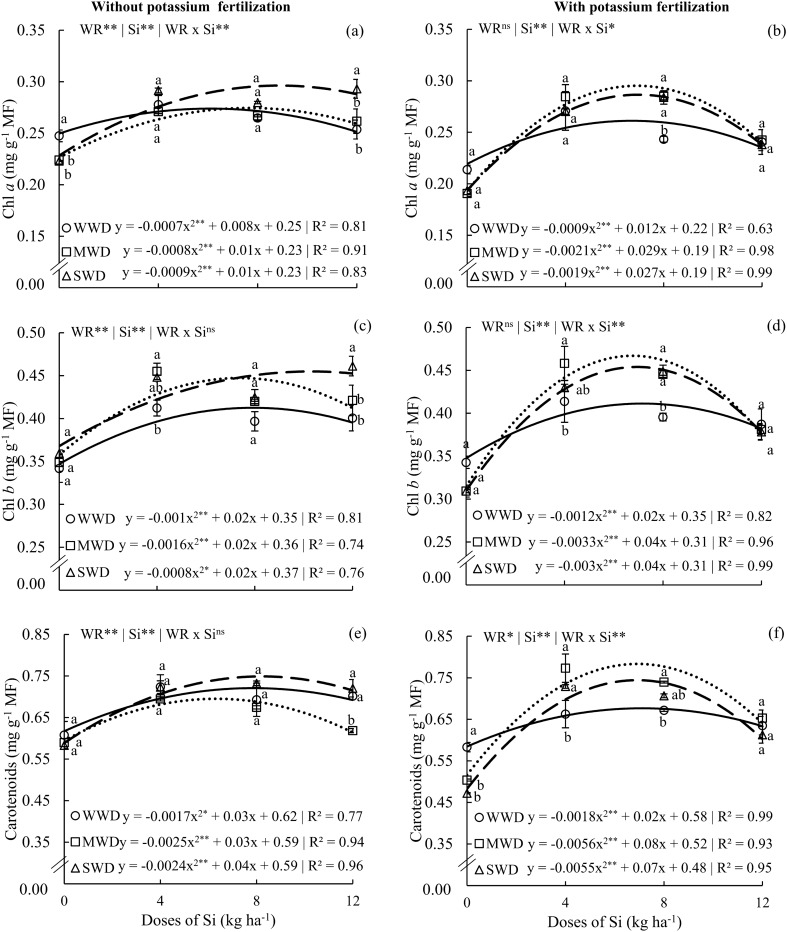
Chlorophyll a content (Chl a) (**a**,**b**), chlorophyll b content (Chl b) (**c**,**d**), and carotenoid content (**e**,**f**) in common bean plants cultivated under well-watered conditions—WWD (80% of water retention capacity—WRC), moderate water deficit—MWD (60% of WRC), and severe water deficit—SWD (40% of WRC), the plants were subjected to four doses of Si (0, 4, 8, and 12 kg ha^−1^) via fertigation, with and without potassium fertilization. Letters indicate significant differences among water regimes (WR) at each Si dose (p < 0.05, Tukey’s test). * and **Indicate significance at the 1% and 5% probability levels, respectively, while ns indicates no significance based on the F test.

The chlorophyll a (Chl*a*) photosynthetic pigments showed interactive effects of water regimes and Si doses in plants without potassium fertilization and with potassium fertilization (Fig. 4a,b). The effect of Si doses resulted in a quadratic polynomial fit for the three water regimes in both potassium fertilization conditions. However, for chlorophyll b (Chl*b*), only plants with K fertilization showed an interactive effect (p < 0.01) (Fig. 4d), following the same regression fitting. Plants without potassium fertilization had maximum Chl*a* contents of 0.27, 0.26, and 0.25 mg g^−1^ at Si doses of 5.7, 6.2, and 5.5 kg ha^−1^ for WWD, MWD, and SWD, respectively (Fig. 4a). Plants that received K fertilization presented maximum levels of Chl*a* of 0.26, 0.29, and 0.28 mg g^−1^ at doses of 6.6, 6.9, and 7.1 kg Si ha^−1^ (Fig. 4b). In plants without fertilization, with K and without Si (0 kg Si ha^−1^), plants under WWD had higher Chl*a* contents compared to plants under MWD and SWD. However, at the highest dose of Si (12 kg ha^−1^), plants under SWD had a higher content of this pigment.

The maximum Chl*b* contents were 0.45, 0.42, and 0.49 mg g^−1^ at Si doses of 10.0, 6.2, and 12.5 kg ha^−1^ of Si in plants without potassium fertilization (Fig. 4c). In plants with potassium fertilization, the maximum levels of Chl*b* were 0.43, 0.43, and 0.44 mg g^−1^ obtained at doses of 8.3, 6.0, and 6.6 kg Si ha^−1^ for WWD, MWD, and SWD, respectively (Fig. 4d). Plants without potassium fertilization and with potassium fertilization under MWD showed a higher Chl*b* content at the dose of 4 kg Si ha^−1^. However, at the highest dose of Si (12 kg ha^−1^), only plants under SWD showed a higher content of this pigment. In plants with potassium fertilization, water deficit, whether moderate or severe, increased the Chl*b* content only at the dose of 8 kg ha^−1^.

The carotenoid contents of common bean plants without potassium fertilization were influenced by the factors in an isolated manner (p < 0.01) (water regime and Si doses), while in plants with potassium fertilization, there was an effect of the interaction between factors (WR x Si) (p < 0.01). Both K conditions presented a quadratic polynomial fit of water regimes according to increasing doses of Si (Fig. 4e,f). Plants without potassium fertilization showed a maximum content of leaf carotenoids of 0.75, 0.68, and 0.76 mg g^−1^ at Si doses of 8.8, 6.0, and 8.3 kg ha^−1^ for WWD, MWD, and SWD, respectively (Fig. 4e). Plants with potassium fertilization obtained maximum carotenoid contents of 0.63, 0.80, and 0.70 mg g^−1^ at doses of 5.5, 7.1, and 6.3 kg Si ha^−1^ for WWD, MWD, and SWD, respectively (Fig. 4f). In this K condition, the intermediate doses of Si (4 and 8 kg ha^−1^) in plants under MWD and SWD presented higher carotenoid contents compared to plants cultivated under WWD.

**Figure 5:**
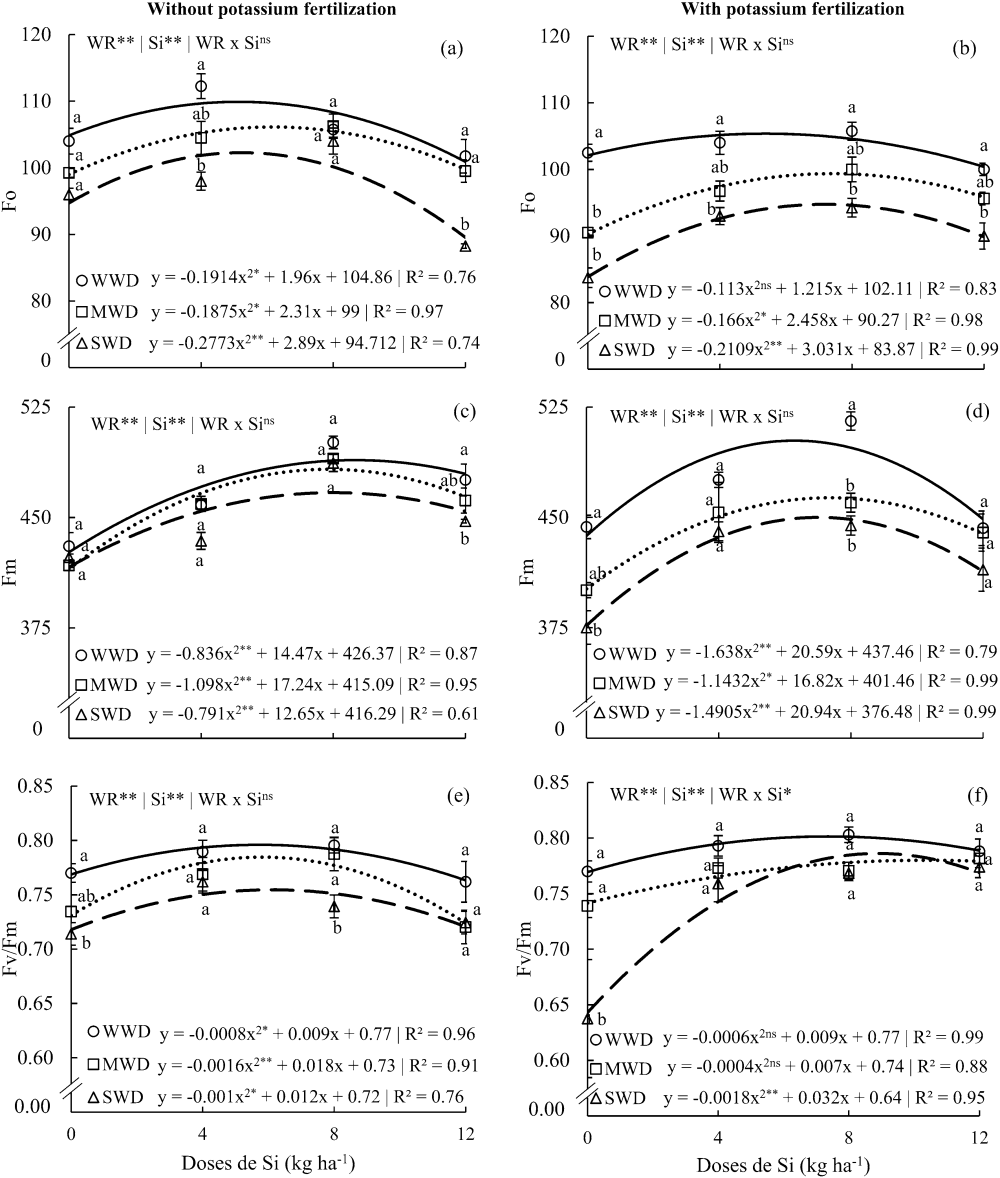
Initial fluorescence (Fo) (**a**,**b**), maximum fluorescence (Fm) (**c**,**d**), and quantum efficiency of photosystem II (Fv/Fm) (**e**,**f**) in common bean plants cultivated without water deficit (WWD, 80% of the water retention capacity—WRC), with moderate water deficit (MWD, 60% of WRC), and with severe water deficit (SWD, 40% of WRC), the plants were subjected to four doses of Si (0, 4, 8, and 12 kg ha^−1^) via fertigation, with and without potassium fertilization. Letters indicate significant differences among water regimes (WR) at each Si dose (p < 0.05, Tukey’s test). * and **Indicate significance at the 1% and 5% probability levels, respectively, while ns indicates no significance based on the F test.

The isolated factors, water regime, and Si doses, affected Fo and Fm in all three water regimes and both potassium fertilization conditions (Fig. 5a,d), as well as Fv/Fm in plants without potassium fertilization (Fig. 5e). The interaction effect (WR x Si) was significant only for Fo and Fv/Fm with potassium fertilization (p < 0.05) (Fig. 5f).

The values of Fo, Fm, and Fv/Fm in plants without potassium fertilization and with potassium fertilization showed a quadratic polynomial fit with applied Si doses (Fig. 5a,f). The maximum values in plants without potassium fertilization were 109.8, 106.1, and 102.2 for Fo at doses of 5.1, 6.1, and 5.2 kg Si ha^−1^; 488.9, 482.7, and 466.9 for Fm at doses of 8.6, 7.8, and 7.9 kg Si ha^−1^; and 0.79, 0.78, and 0.75 for Fv/Fm at doses of 5.6, 5.6, and 6.0 kg Si ha^−1^ for the WWD, MWD, and SWD water regimes, respectively. For plants that received potassium fertilization, the maximum results were 105.3, 99.3, and 94.7 for Fo with doses of 5.3, 7.4, and 7.1 kg Si ha^−1^; 502.1, 463.3, and 450.0 for Fm with doses of 6.2, 7.3, and 7.0 kg Si ha^-1^; and 0.804, 0.771, and 0.782 for Fv/Fm with doses of 7.5, 8.7, and 8.8 kg Si ha^−1^. Plants under SWD without receiving Si via fertigation showed lower Fv/Fm values under both K conditions. However, only at the dose of 8 kg Si ha^−1^, plants under MWD without potassium fertilization did not differ from plants under SWD (Fig. 5a).

**Figure 6:**
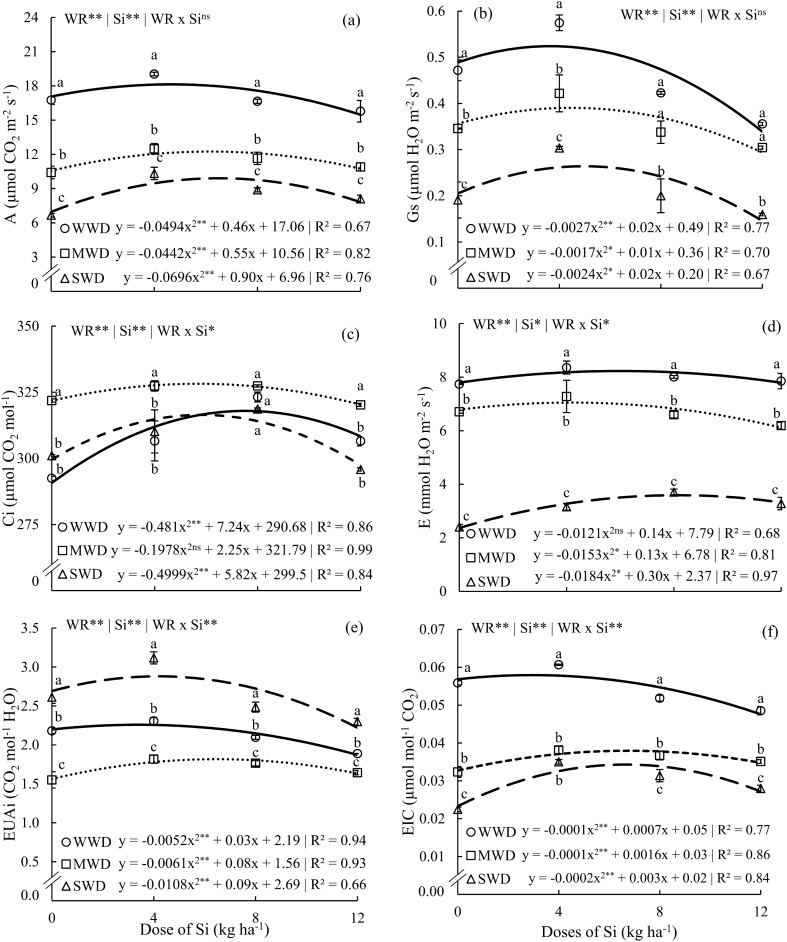
Net photosynthesis (A) (**a**), stomatal conductance (Gs) (**b**), internal C concentration (Ci) (**c**), transpiration rate (E) (**d**), Intrinsic water use efficiency (WUE) (**e**), and instantaneous efficiency of carboxylation (EIC) (f) in common bean plants cultivated without water deficit (WWD, 80% of the water retention capacity—WRC), with moderate water deficit (MWD, 60% of WRC), and with severe water deficit (SWD, 40% of WRC), the plants were subjected to four doses of Si (0, 4, 8, and 12 kg ha^−1^) via fertigation, without potassium fertilization. Letters indicate significant differences among water regimes (WR) at each Si dose (p < 0.05, Tukey’s test). * and **Indicate significance at the 1% and 5% probability levels, respectively, while ns indicates no significance based on the F test.

Photosynthesis (A) and stomatal conductance (Gs) of common bean plants showed significant effects for the isolated factors (p < 0.01). The results of Si doses were adjusted to the quadratic polynomial regression model (Fig. 6a,b). The internal concentration of CO_2_ (Ci) (p < 0.05), transpiration (E) (p < 0.05), water use efficiency (WUEi) (p < 0.01), and carboxylation efficiency (EIC) (p < 0.01) were influenced by the effects of the interaction between factors (WR x Si). However, they also followed a quadratic polynomial model (Fig. 6c–f).

The maximum values of A were 18.1, 12.2, and 9.8 µmol CO_2_ m^−2^ s^−1^ obtained at the doses of 4.6, 6.2, and 6.5 kg Si ha^−1^ for conditions under WWD, MWD, and SWD, respectively. At all Si doses, plants under SWD had lower A values, while plants under WWD had the highest values for this variable (Fig. 6a). For Gs, the maximum values were 0.52, 0.37, and 0.24 µmol H_2_O m^−2^ s^−1^ at the doses of 3.7, 2.9, and 4.1 kg Si ha^−1^ in plants under WWD, MWD, and SWD, respectively. At the lowest Si doses (0 and 4 kg Si ha^−1^), plants under WWD showed higher rates of Gs compared to those subjected to MWD and SWD conditions. At doses of 8 and 12 kg Si ha^−1^, plants under WWD and MWD showed Gs rates similar to each other and higher than those under SWD (Fig. 6b).

The highest concentrations of CO_2_ were 317.9, 328.1, and 316.4 µmol CO_2_ mol^−1^ at Si doses of 7.5, 5.6, and 5.8 kg Si ha^−1^ for WWD, MWD, and SWD, respectively. Plants under MWD showed higher Ci at doses of 0, 4, and 12 kg Si ha^-1^ compared to plants under WWD and SWD. However, at the dose of 8 kg ha^−1^ of Si, the three water regimes presented similar Ci (Fig. 6c).

The maximum rates of E occurred in plants that received doses of 5.7, 4.2, and 8.1 kg Si ha^−1^, reaching values of 8.1, 7.0, and 3.5 nmol H2O m^−2^ s^−1^ for WWD, MWD, and SWD, respectively. The highest E was obtained in plants grown under WWD. However, the lowest E was obtained in plants under SWD at all Si doses supplied (Fig. 6d).

The Si doses that provided the maximum WUEi were 2.2, 1.8, and 2.8 CO_2_ mol^−1^ H_2_O at doses of 2.8, 6.5, and 4.1 kg Si ha^−1^ for WWD, MWD, and SWD, respectively. For the EIC variable, the maximum values obtained were 0.051, 0.036, and 0.031 µmol mol^−1^ CO_2_ for doses of 3.5, 8.0, and 7.5 kg Si ha^−1^ under WWD, MWD, and SWD, respectively (Fig. 6e,f). Water use efficiency and carboxylation efficiency were higher in plants under SWD and WWD, respectively, at all Si doses, compared to plants under WWD.

### Silicon effects in dry mass content of common beans

**Figure 7:**
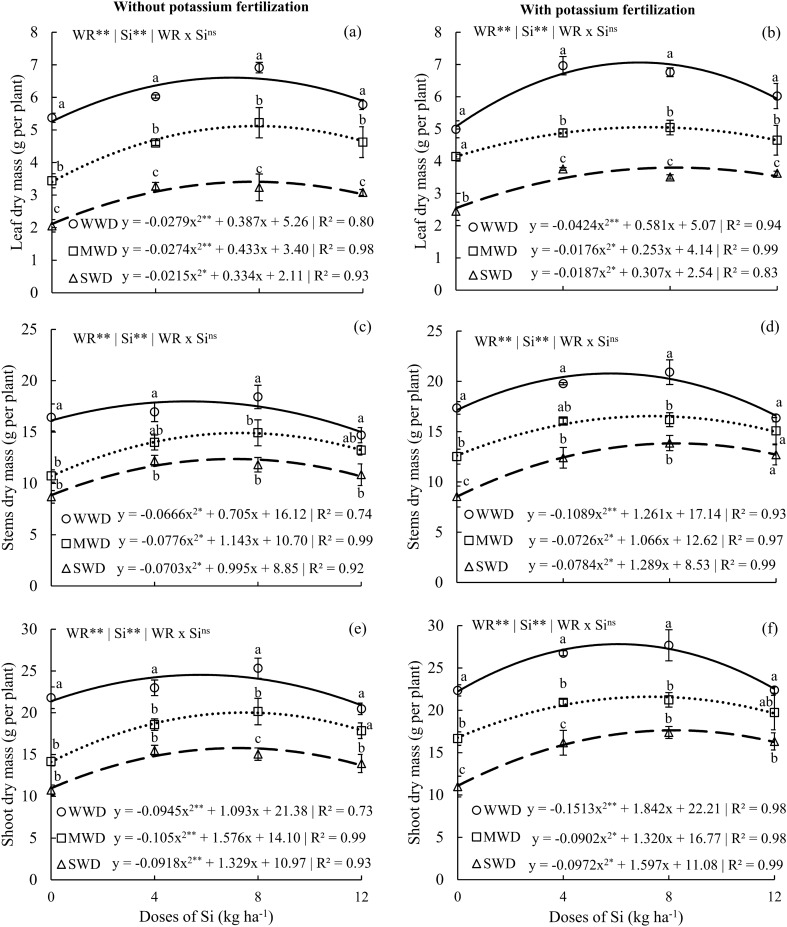
Leaf dry mass (**a**,**b**), stem dry mass (**c**,**d**), and shoot dry mass (e, f) in common bean plants cultivated without water deficit (WWD, 80% of the water retention capacity—WRC), with moderate water deficit (MWD, 60% of WRC), and with severe water deficit (SWD, 40% of WRC), the plants were subjected to four doses of Si (0, 4, 8, and 12 kg ha^−1^) via fertigation, with and without potassium fertilization. Letters indicate significant differences among water regimes (WR) at each Si dose (p < 0.05, Tukey’s test). * and **Indicate significance at the 1% and 5% probability levels, respectively, while ns indicates no significance based on the F test.

Leaf dry mass, stem dry mass, and shoot dry mass of plants without potassium fertilization and with potassium fertilization had isolated effects of the factors water regime and doses of Si (p < 0.01). For the production of dry mass, the Si doses provided an effect with a quadratic polynomial fit for all water regimes in both potassium fertilization conditions (Fig. 7a,f).

The results for the maximum production of leaf dry mass in plants without potassium fertilization were 6.6, 5.1, and 3.4 g per plant obtained with Si doses of 6.9, 7.9, and 7.7 kg Si ha^-1^; for plants that received potassium fertilization, the maximum values were 7.0, 5.0, and 3.8 g per plant obtained at the doses of 6.8, 6.6, and 8.2 kg Si ha^-1^ for WWD, MWD, and SWD, respectively (Fig. 7a,b). Plants in the WWD regime had higher leaf dry mass than those submitted to the MWD and SWD regimes at Si doses of 4, 8, and 12 kg ha^−1^ in both K conditions.

The results for the maximum stem dry mass production in plants without potassium fertilization were 17.9, 14.9, and 12.6 g per plant; with potassium fertilization, they were 20.7, 16.5, and 13.8 g obtained at Si doses of 5.2, 7.3, and 7.0; 5.7, 7.3, and 8.2 kg ha^−1^ for the WWD, MWD, and SWD regimes, respectively (Fig. 7c,d). Plants that did not receive potassium fertilization but were fertilized with Si doses of 4 and 12 kg ha^−1^ in the MWD water regime did not differ from plants under WWD (Fig. 7c). However, in plants with potassium fertilization, this result also occurred for SWD at the highest dose of Si (12 kg ha^−1^) (Fig. 7d).

The results for the maximum production of shoot dry mass in plants without potassium fertilization were 24.5, 20.0, and 15.7 g per plant; in plants that received potassium fertilization, the values were 27.8, 21.5, and 17.6 g per plant, at Si doses of 5.7, 7.5, and 7.2, 6.0, 7.3, and 8.2 kg ha^−1^ for the WWD, MWD, and SWD, respectively (Fig. 7e,f). At the dose of 12 kg ha^−1^, plants cultivated under MWD did not differ from plants cultivated under WWD in the condition without potassium fertilization. Plants under SWD with a supply of 4 kg Si ha^−1^ showed a similar dry mass to that of plants under MWD (Fig. 7e). On the other hand, plants fertilized with K and under SWD, but which received Si at higher doses (8 and 12 kg ha^−1^), showed the same dry mass as that of plants under MWD (Fig. 7f).

### Correlations and heat map

**Figure 8:**
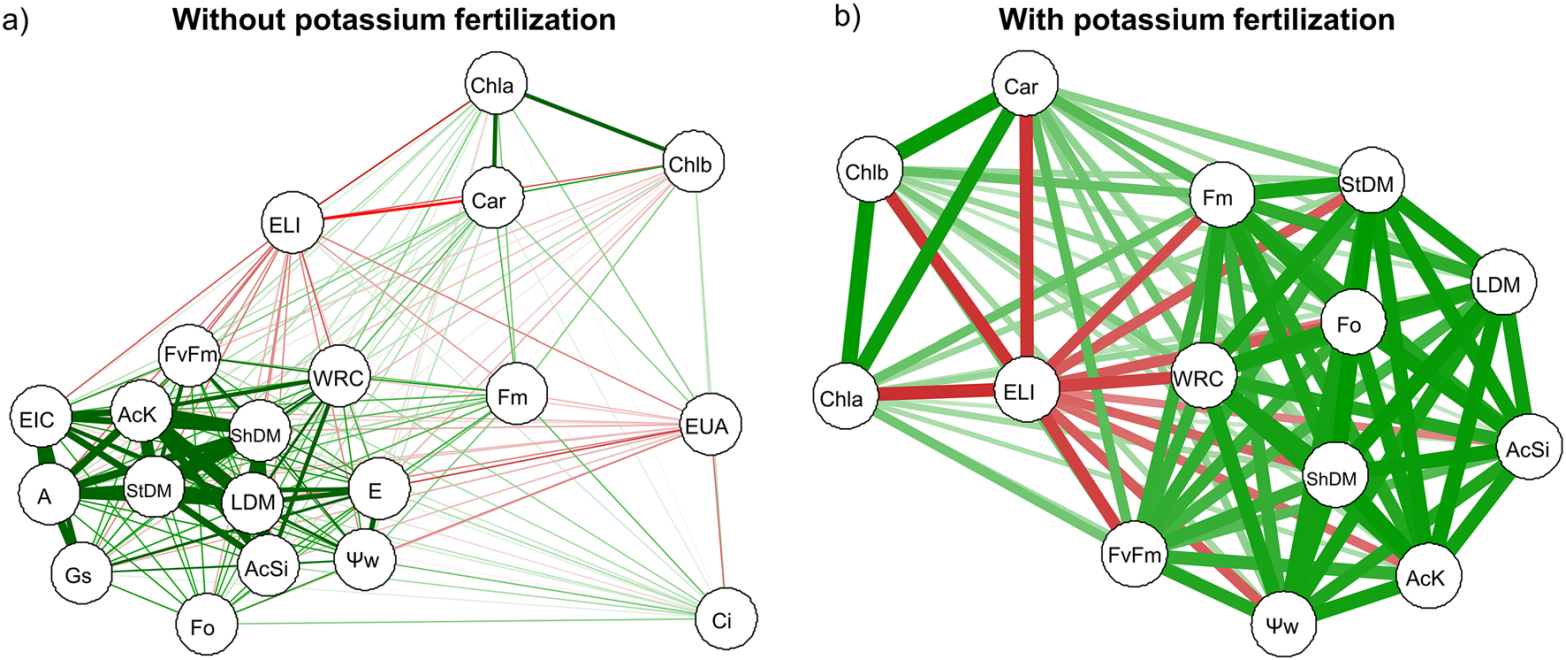
Pearson correlation network between the variables silicon accumulation (AcSi) and potassium (AcK), relative water content (WRC), leaf water potential (Ψw), electrolyte leakage (ELI), chlorophyll a content (Chl*a*), chlorophyll b content (Chl*b*), and carotenoid content (Car), initial fluorescence (Fo), maximum fluorescence (Fm), quantum efficiency of photosystem II (Fv/Fm), leaf dry mass (LDM), stem dry mass (StDM), and aboveground dry mass (ShDM) in common bean plants cultivated without (**a**) and with (**b**) potassium fertilization; and photosynthesis (A), stomatal conductance (Gs), intercellular CO_2_ concentration (Ci), transpiration (E), water use efficiency (WUE), and intrinsic carboxylation efficiency (EIC) in common bean plants cultivated without water deficit (WWD, 80% of the water retention capacity—WRC), with moderate water deficit (MWD, 60% of WRC), and with severe water deficit (SWD, 40% of WRC), the plants were subjected to four doses of Si (0, 4, 8, and 12 kg ha^−1^). The positive correlations were highlighted in green, and the negative correlations were represented in red; the thickness of the line indicates the strength of the correlation.

The Pearson correlation revealed that in plants that not received potassium fertilization, the variable AcSi (silicon accumulation) showed a strong positive correlation with LDM (leaf dry mass) (r = 0.92), ShDM (stem dry mass) (r = 0.91), StDM (shoot dry mass) (r = 0.90), AcK (potassium accumulation) (r = 0.92), EIC (instantaneous carboxylation efficiency) (r = 0.76), A (net assimilation rate) (r = 0.80), and Gs (stomatal conductance) (r = 0.66). On the other hand, Fo (initial fluorescence) (r = 0.65), Fv/Fm (maximum quantum yield of photosystem II) (r = 0.82), E (transpiration rate) (r = 0.73), Ψw (water potential) (r = 0.74), WRC (water retention capacity) (r = 0.88), Fm (maximum fluorescence) (r = 0.65), Ci (intercellular CO2 concentration) (r = 0.28), Car (carotenoids) (r = 0.27), Chl*a* (chlorophyll a) (r = 0.11), and Chl*b* (chlorophyll b) (r = 0.07) exhibited positive correlations but with a lower intensity. Additionally, the WUE (water use efficiency) (r = − 0.22) had a negative correlation with ELI (Electrolyte Leak Index) (r = − 0.38) (Fig. 8a). In plants with potassium fertilization, AcSi showed positive correlations with AcK (r = 0.93), LDM (r = 0.95), StDM (r = 0.96), Fm (r = 0.87), Fo (r = 0.96), ShDM (r = 0.97), Ψw (r = 0.88), and WRC (r = 0.86), as well as with Car (r = 0.31), Chl*a* (r = 0.25), and Chl*b* (r = 0.25). Conversely, ELI (r = − 0.48) exhibited a negative correlation with these variables (Fig. 8b).

**Figure 9:**
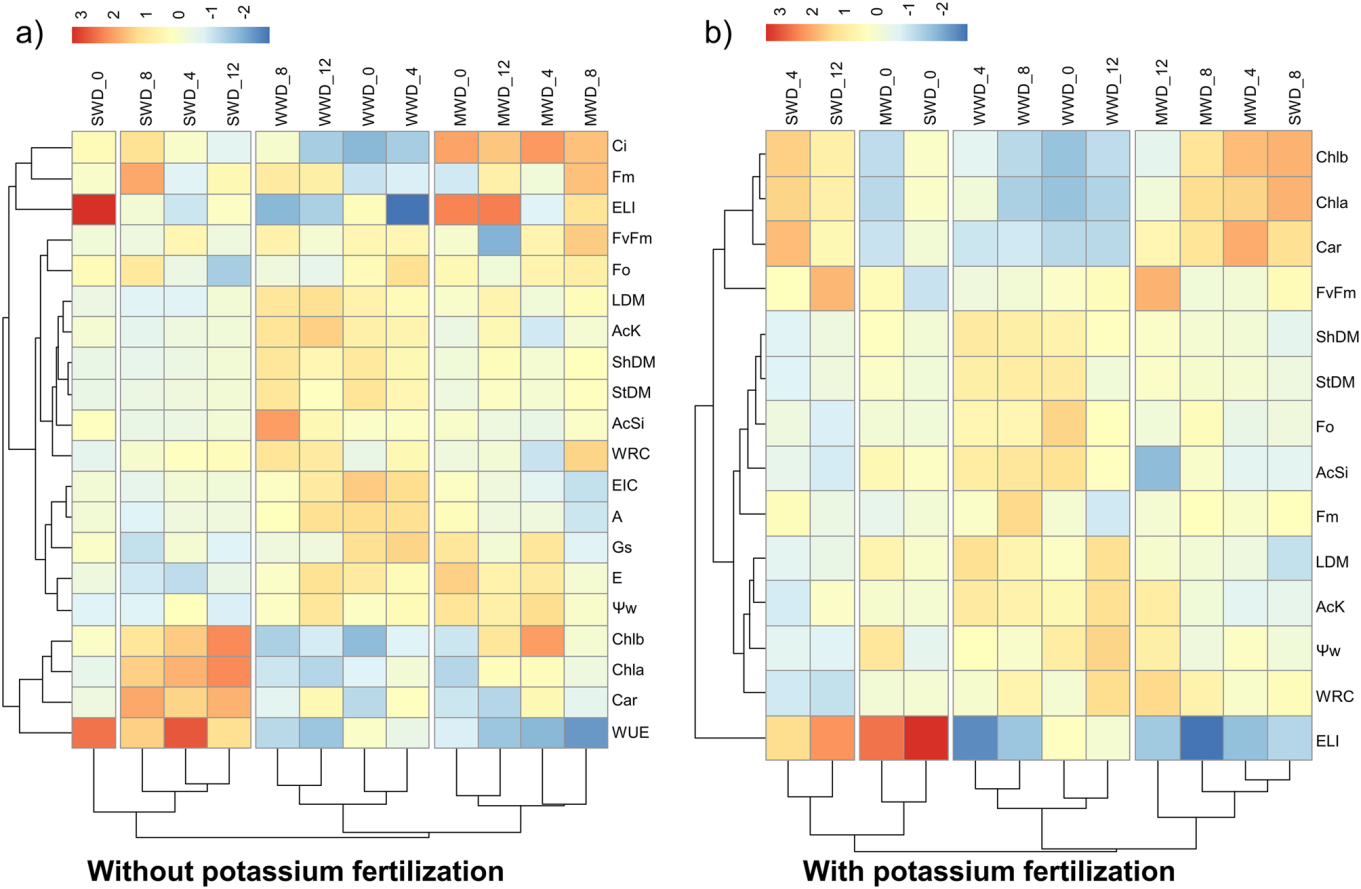
Hierarchical cluster analysis of the data for silicon accumulation (AcSi) and potassium (AcK), relative water content (WRC), water potential (WaterPotential), electrolyte leakage index (ELI), chlorophyll a content (Chl*a*), chlorophyll b content (Chl*b*), and carotenoid content (Car), initial fluorescence (Fo), maximum fluorescence (Fm), quantum efficiency of photosystem II (Fv/Fm), leaf dry mass (LDM), stem dry mass (StDM), and aboveground dry mass (ShDM) in common bean plants cultivated without (**a**) and with (**b**) potassium fertilization; and photosynthesis (A), stomatal conductance (Gs), intercellular CO2 concentration (Ci), transpiration (E), water use efficiency (WUE), and intrinsic carboxylation efficiency (EIC). In both experiments (without and with potassium fertilization), the plants were subjected to three water conditions: well-watered—WWD (80% of water retention capacity—WRC), moderate water deficit—MWD (60% of WRC), and severe water deficit—SWD (40% of WRC); combined with doses of Si provided through fertigation: 0, 4, 8, and 12 kg ha^−1^.

In the cluster analysis, it was observed that in the condition without potassium fertilization, the WUE was directly related to the WRC and ELI, which were further associated with the other analyzed variables in the doses of 4 to 8 kg Si ha^−1^ under severe water regime (Fig. 9a). However, in plants that received potassium fertilization, the ELI was directly related to Fv/Fm (maximum quantum yield of photosystem II), carotenoids, and leaf dry mass, while the rest of the analyzed variables were particularly associated with the moderate and sufficient water regimes in the doses of 4 to 8 kg Si ha^−1^ (Fig. 9b).

## Discussion

### Application via fertigation improves its absorption and increases the accumulation of potassium in common beans.

The positive effect of Si in mitigating abiotic stresses has primarily been demonstrated in research conducted on plants of the Poaceae family, which have a high capacity for Si accumulation in their tissues due to efficient Si transporters in their cell membranes^47^. However, this study focused on a species from the legume group, which has limited Si accumulation capacity and maintains a higher Si content in the roots compared to the shoots^23^. Reports on the effect of Si on plants with low Si uptake potential are limited to a few species such as tomato and cucumber^48,49^. Furthermore, these studies are often conducted in pots and may not fully represent field crop conditions.

In this study, we conducted the first field-level investigation with common bean plants subjected to Si application via fertigation under different water regimes. The results of this study indicate that although common bean is classified as a non-Si-accumulating species^50^, the supply of Si via fertigation efficiently increases Si accumulation in the plants (Fig. 2a,b). This finding is significant as it raises the expectations for crop responses to Si.

The efficiency of Si application can be attributed to proper handling and the initial use of a low-concentration fluid source stabilized with sorbitol, which is below the level of silicate polymerization^16^. This facilitates the root absorption of Si by the plant. The use of soluble Si sources presents advantages over insoluble and solid sources, which have slower reactions and require larger amounts to be incorporated into the soil^15,51^. Therefore, the effects of Si observed in this study demonstrate, for the first time, that Si application via fertigation, especially at doses resulting in higher Si enrichment in the plant, occurs at relatively low doses (< 10 kg Si ha^−1^), both in the absence and presence of potassium fertilization.

The optimal Si doses increased Si accumulation in common beans, both without the addition of K and with the addition of K, with greater prominence observed in plants under an adequate water regime (WWD) (96% and 29% increase) compared with plants to moderate water deficit (MWD) (95% and 39% increase) and severe water deficit (SWD) (77% and 71% increase), respectively. The high Si accumulation demonstrates the excellent Si absorption capacity of common bean in a fertigated system. These results are consistent with findings reported for other species, such as sugarcane^51^. This finding offers an efficient Si addition alternative for irrigated crops at the field level, including species from the legume group, as it enables significant Si absorption at relatively low doses. This has the potential to expand the use of Si in irrigated agriculture.

It is important to note that the increase in K accumulation in common bean plants was evident in plants that received potassium fertilization compared to those without it (Fig. 2c,d). However, a novel finding was that Si supply increased the accumulation of K in both potassium conditions, observed in all three water regimes, particularly in plants under WWD. This can be explained because the presence of silicon stimulates vacuolar redistribution through potassium root transporters to the plant shoots^52^, as reported for corn plants^15,53^ and beans in hydroponic systems^23^.

### Silicon improves the water status of tissues and is reflected in photosynthetic parameters

Plants without Si addition was evidently affected by moderate or severe water stress, resulting in a decrease in relative water content compared to plants with sufficient water (Fig. 3a,b). However, the addition of Si efficiently reversed the damage caused by moderate and severe water deficits, maintaining the hydration of plant tissues under both potassium fertilization conditions, as evidenced by the adjustment of leaf water potential (Fig. 3c,d). Si absorbed by plants is deposited in the cuticle and guard cells, forming a silica layer that reduces water loss during transpiration^24,47^. Si supply improves the root's ability to absorb water by enhancing root osmotic potential and influencing the activation of aquaporins involved in water transport, thereby facilitating water flow in the xylem^48,54^.

Under water deficit conditions, plants experience cellular dehydration, which promotes the formation and excessive accumulation of reactive oxygen species, leading to cell membrane degradation. In this study, we observed an increase in electrolyte leakage, indicating cellular degradation, in plants subjected to severe and moderate water deficits. However, the addition of Si partially mitigated this effect compared to plants without Si supply. The decrease in electrolyte leakage rate with Si application has been previously observed in sugarcane^14^, corn^15^ and fava bean^24^.

The beneficial effect of Si in reducing electrolyte leakage in plants experiencing water deficit is crucial as it preserves the integrity of cellular structures, leading to a higher content of photosynthetic pigments, particularly chlorophylls and carotenoids, in plants subjected to moderate and severe water deficiencies under both potassium conditions (Fig. 4). The high concentration of carotenoids present in the leaves (Fig. 4e,f) contributes to the photosynthetic efficiency of plants with and without water deficit (Fig. 8). Carotenoids play a protective role by helping to eliminate reactive oxygen species and dissipating excess energy generated in photosystems, thereby improving the quantum efficiency of photosystem II (Fv/Fm)^15,55^. Photosystem II is responsible for the variable fluorescence of chlorophyll *a*^56,57^. The preservation of chlorophyll and carotenoid contents demonstrates that plants receiving potassium fertilization along with Si applications via fertigation can maintain the levels of photosynthetic pigments, especially under water deficiency conditions.

Thus, by preserving the integrity of leaf pigments, Si acts as an attenuating agent for moderate and severe water deficiency, improving initial (Fo) and maximum (Fm) fluorescence, as well as Fv/Fm, which could have been adversely affected in plants under the same water conditions but without Si. Similar results have been observed in barley^57^, particularly in Fo (Fig. 5a,b) and Fm (Fig. 5c,d), showing a greater effect when Si was supplied under severe and moderate water stress conditions in both potassium conditions. Similar effects have been reported for common bean plants under less severe stress conditions and with the application of salicylic acid^58^. Fv/Fm was also influenced by water deficit, but the application of an appropriate Si dose was essential to enhance the photosynthetic efficiency of plants (Fig. 5e,f). Similar findings have been reported for other species, such as forage plants^19^ and sugarcane^18^.

Enrichment of Si in plants favored gas exchange, particularly the photosynthetic rate, transpiration, and stomatal conductance, under water deficit conditions (Fig. 6a,b,d), which is consistent with the effects observed in fava beans^24^. Plants cultivated under a severe water regime exhibited higher water use efficiency compared to plants under adequate and moderate water deficit regimes (Fig. 6e). Si supply has also been found to increase water use efficiency under water stress conditions in wheat and sorghum plants^12,59^. Regarding Ci (Fig. 6c), Si application did not significantly, showing a negative correlation with water use efficiency (Fig. 8a), differ from what has been reported for sorghum crops, where Ci remained constant regardless of Si supply under both adequate and deficient water conditions^12^. In a previous study with common beans under water deficit without Si addition, these values experienced a significant decrease of 75% in gas exchange^60^, However, in this study, the addition of Si improved these parameters by up to 40%. These results demonstrate that Si is an alternative for common bean cultivation as it enhances these physiological parameters. The supply of Si via fertigation was highly effective in providing plants with this element. Thus, we confirm our first hypothesis that Si promotes gas exchange, photosynthetic pigments, water potential, and relative water content, while maintaining membrane integrity in irrigated systems with different soil water contents.

The physiological parameters evaluated in common beans were enhanced by the use of Si at an average dose close to 6.9 kg ha^−1^, regardless of the potassium condition. Higher Si doses did not further improve physiological processes in the plants, likely due to the polymerization of Si resulting from increased deposition of the element in roots in the form of amorphous silica^61,62^ This may be attributed to decreased Si absorption at higher doses.

The results of this research demonstrate that the beneficial effects of Si on the physiological aspects studied are not limited by potassium, as positive effects were observed in both potassium conditions. This is likely because there was no severe potassium limitation in the plants in the treatment without potassium application, as the potassium content in the soil of this experiment is not limited (K = 6.2 mmol_c_ dm^−3^ at the 0–20 cm layer)^63^.

### Silicon application improved the accumulation of dry matter in common beans

The growth of common bean plants was limited under moderate and severe water deficit without Si supply, resulting in low shoot mass production, consistent with findings reported by other authors^64^. However, with the supply of Si at the optimal dose compared to the zero dose, there was an increase in shoot dry mass production in both experiments (with and without potassium), with increases of 41% and 54% (MWD), 47% and 60% (SWD), and 12% and 24% (WWD), respectively. The lack of interaction between Si doses and water regimes in the dry mass production of different plant organs indicates that the beneficial effect of Si is independent of soil water content. Therefore, the beneficial effect of Si on common bean growth is significant under water stress conditions but to a lesser extent under well-watered conditions. These findings differ from indications that the effects of Si on non-accumulating plants are limited to stress crops, as observed in potato plants^65^ and other species^27,66^.

This study provides the first report that common bean plants cultivated in the field, which have only passive Si uptake mechanisms, benefit from the stress-attenuating effect promoted by Si in both water-deficient and water-sufficient regimes. Thus, we confirm the second hypothesis, which suggests that the benefits of Si may occur in both water-deficient and water-sufficient regimes.

Therefore, we have shown that the benefit of Si via fertigation is consistent across areas with different soil water and potassium contents. This extends the recommendation of Si for various agricultural management practices; however, the optimal Si dose may vary. We have also discovered the need to use a higher Si dose in water-deficient regimes (7.2–8.2 kg Si ha^−1^) due to lower Si absorption compared to the sufficient water regime (5.7–6.0 kg Si ha^−1^), which maximizes dry matter production under both potassium conditions. Furthermore, we confirm the third hypothesis, indicating that there is an optimal Si dose, which may be higher in water-deficient regimes compared to the sufficient water regime, regardless of potassium conditions. Therefore, the initial general recommendation for Si in common bean cultivation is to apply relatively low Si doses ranging from 5.7 to 8.2 kg ha^−1^, depending on the water availability, as it constitutes a useful and sustainable strategy for improving water use.

The broad beneficial effect of Si on common beans cultivated in the field, a species sensitive to water deficit^67^, has global implications as it can benefit regions where this species is cultivated in years with abundant water availability or during years with water restrictions, necessitating the adoption of controlled deficit irrigation systems. Controlled deficit irrigation systems were initially proposed by Mitchell^68^ and may be a viable strategy considering the projected worsening of climate change, which has altered rainfall patterns worldwide^6,7^. This research, focusing on strategies to improve common bean growth, can also benefit low-income populations that rely on a vegetarian diet, as beans are commonly referred to as the "poor man's source of protein" (> 20% protein)^69^.

Future research should further expand and elucidate the effects of Si on common beans, a species highly responsive to water supply. It is opportune to study additional mechanisms, such as nutritional and molecular aspects, to gain a comprehensive understanding of the benefits provided by Si.

## Conclusion

Si application via fertigation improves plant conditions under severe water stress, regardless of potassium status. This improvement is evident in terms of relative water content, leaf water potential, and membrane resistance, which directly impact pigments and gas exchange. The physiological effects of Si application enhance photosynthesis and mitigate the negative effects of water deficit stress. These findings demonstrate, for the first time in common beans the potential of Si for improving irrigation efficiency in irrigated crops in fields conditions, but its needs more investigations futures with specie.

### Supplementary Information

Supplementary Information.

## Data Availability

All data generated or analysed during this study are included in this manuscript.
