# Supplementary material for: Silicon via fertigation with and without potassium application, improve physiological aspects of common beans cultivated under three water regimes in field
Source: Sci Rep. 2024 Jan 24;14:2051. doi: 10.1038/s41598-024-52503-8 (PMC10808205; doi:10.1038/s41598-024-52503-8)
Supplement: Supplementary file 1 — Supplementary Information. [file 41598_2024_52503_MOESM1_ESM.pdf]

Table1. Climate history of the last 30 years

Years	Min. Temp. (°C)	Max. Temp. (°C)	Med. Temp. (°C)	R.H. (%)	Radiation Global (MJ m^{-2})	Precipitation Total (mm)
1992	17,1	28,3	21,9	75,80	-	1411,00
1993	17,1	29,1	22,2	73,53	-	1710,50
1994	17,1	30,2	22,7	68,12	-	1055,60
1995	17,4	29,7	22,6	70,00	-	1552,10
1996	16,8	29,2	22,1	72,13	-	1286,30
1997	17,0	29,5	22,3	71,59	-	1429,40
1998	17,3	29,6	22,6	73,77	-	1291,70
1999	16,6	29,9	22,2	68,76	-	1526,50
2000	16,9	29,7	22,6	69,67	-	1207,80
2001	17,1	29,7	22,6	70,68	-	1265,40
2002	17,7	30,4	23,1	68,46	-	1536,20
2003	17,0	30,0	22,5	69,40	-	1262,30
2004	16,9	29,2	22,0	71,58	-	1519,10
2005	17,5	29,7	22,6	72,05	-	1230,20
2006	17,2	29,7	22,5	70,58	-	1447,20
2007	17,4	30,0	22,8	69,68	18,75	1585,70
2008	17,1	29,5	22,2	72,35	18,12	1412,40
2009	17,7	29,4	22,5	76,11	17,65	1735,70
2010	17,0	30,2	22,6	70,24	19,28	1225,00
2011	17,0	29,7	22,4	70,07	17,69	1615,70
2012	17,3	30,0	22,8	69,60	17,86	1179,10
2013	17,1	29,4	22,2	72,51	17,68	1453,40
2014	17,3	30,8	23,1	66,48	-	814,60
2015	18,1	30,2	23,1	72,84	-	1711,90
2016	17,2	30,1	22,6	70,60	-	1610,90
2017	17,3	30,1	22,8	67,58	19,51	1207,20
2018	17,6	30,4	23,0	67,76	18,70	1118,50
2019	17,9	30,8	23,5	67,59	19,19	1129,70
2020	17,3	31,0	23,3	64,08	18,86	1156,00
2021	17,1	30,7	23,2	63,27	18,96	1050,90
2022	17,1	30,0	22,7	66,96	18,48	1323,60
**General average**	**17,2**	**29,9**	**22,6**	**70,12**	**18,52**	**1356,83**

(-): Interruption of data collection due to failure and lack of equipment. Annual averages for: temperatures, humidity relative and global radiation.

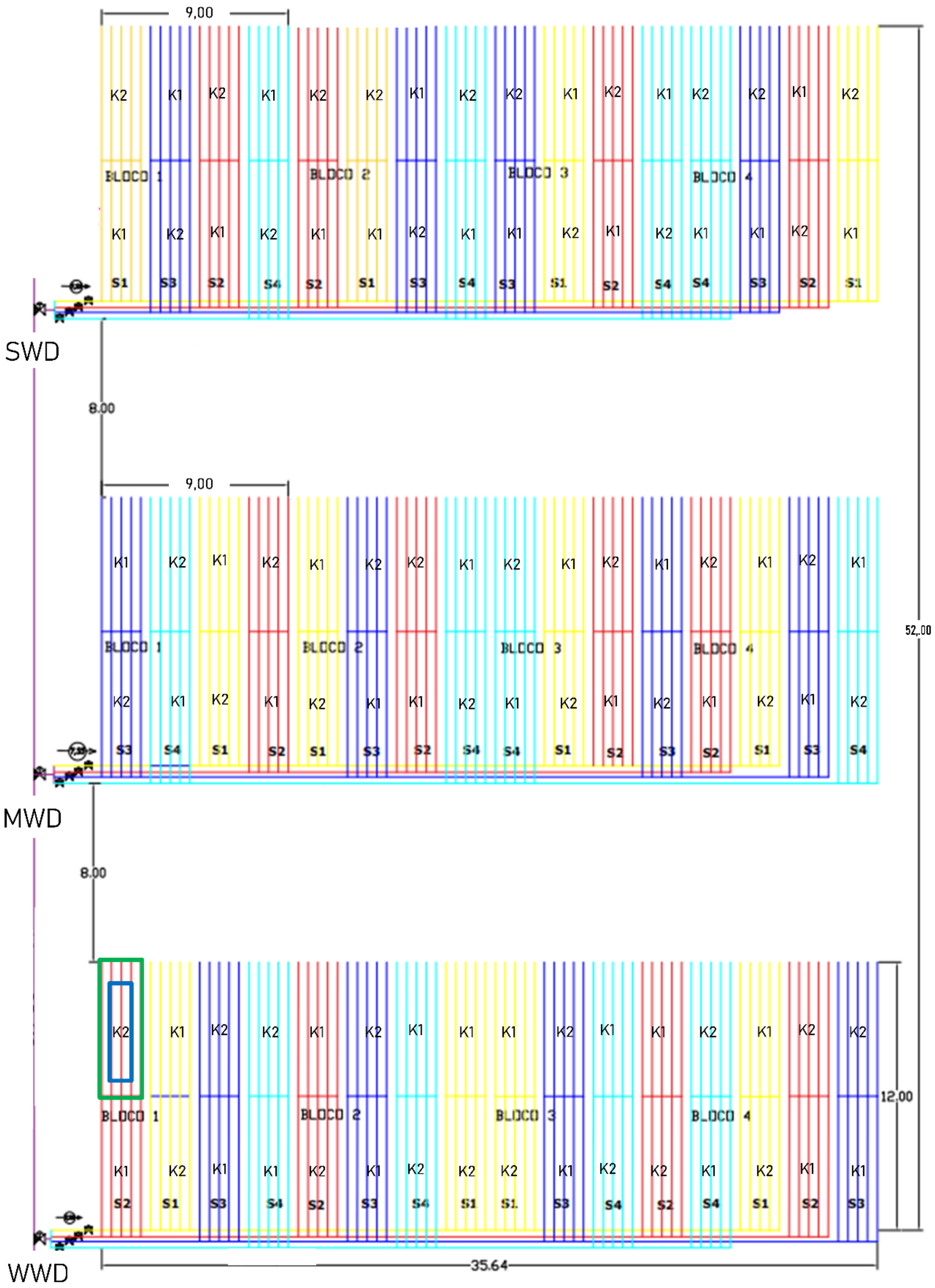

Figure 1. Scheme of the irrigation system and experimental design used in the experiment. Green box represents an experimental plot and the blue box determines the useful area, where the plants were selected for evaluation.
